# Supplementary material for: Association between timed up-and-go test and subsequent pneumonia: A cohort study
Source: PLoS One. 2024 Jan 5;19(1):e0296380. doi: 10.1371/journal.pone.0296380 (PMC10769022; doi:10.1371/journal.pone.0296380)
Supplement: Attachment — Submitted filename: Response letter_revision_20231010.docx [file pone.0296380.s002.docx]

ACADEMIC EDITOR: The remaining comment from the reviewer and editor has not been addressed clearly. Choosing the cutoff score for TUG of 10 s was not justified. You can either justify or calculate the appropriate cutoff score for TUG using the ROC and the AUC because populations differ in some variables such as physical performance.

**Response)** Thank you for your valuable comments. Our study used data from a special health-screening program called the National Screening Program for Transitional Ages (NSPTA) provided by the National Health Insurance Service-Senior Cohort (NHIS-Senior) database of the Korean National Health Insurance (KNHI) service. The NSPTA includes a questionnaire regarding mental/cognitive function and depression screening, as well as physical function tests (TUG test, unipedal balance test) that cover common problems of the elderly, such as frailty.

In the Korean NSPTA, the TUG test cutoff is defined as normal at 10 seconds or less and abnormal at 10 seconds or more based on expert opinion. In addition, the cutoff of 9-10 seconds is used as a cutoff in various populations, and the cutoff of 10 seconds is commonly used in ethnically similar Asian countries such as Japan and Singapore *[Reference- J Am Med Dir Assoc. 2021 Aug;22(8):1640-1645., Phys Ther 2017;97(4):417-24.]* In fact, most Korean studies that have analyzed the association between TUG test and clinical outcomes using data from the National Screening Program for Transitional Ages in Korea have also used a cutoff value of 10 seconds for the TUG *test [Reference- Bone. 2019 Oct;127:474-481., BMC Geriatr . 2020 Mar 19;20(1):111., J Korean Med Sci. 2020 Jan 20; 35(3): e25., J Gerontol A Biol Sci Med Sci. 2018;73(9):1238-43.].*

Furthermore, our study focused on investigating the correlation between a slow TUG result and the occurrence of future pnuemonia, rather than setting a cut off value for the TUG test in detecting pneumonia. Since the cut off suggested by Korean NAPTA and most of the studies using the Korean NAPTA data mentioned above are based on a 10-second cut off, we believe that it is appropriate to apply a 10-second cut off.

Therefore, as you commented, we have further described and written the rationale for setting the cutoff in the Independent variable section as follows. (Line 157-164). We would also like to express our sincere thanks to the editor and reviewers for their helpful comments to improve the paper.

**Materials and methods**

**…**

**Independent variable**

…

*The cut off value of the TUG test varies depending on the study, but the cut off value of 9-10 seconds is used in various population groups (20, 21) , and the cut off value of 10 seconds is also used in Asian populations such as Japan and Singapore (22, 23). In fact, most studies investigating the association of the TUG test with clinical outcomes such as dementia, fractures, and functional disability in Korean populations have used a 10-second cut off value (24-27). Based on these existing studies and expert opinions, the Korean NSPTA defines a TUG test of less than 10 seconds as normal, so in our study, participants were divided into two groups based on their TUG test (<10 seconds as normal and ≥10 seconds as slow gait).*

Reviewers' comments:

Reviewer's Responses to Questions

**Comments to the Author**

1. If the authors have adequately addressed your comments raised in a previous round of review and you feel that this manuscript is now acceptable for publication, you may indicate that here to bypass the “Comments to the Author” section, enter your conflict of interest statement in the “Confidential to Editor” section, and submit your "Accept" recommendation.

Reviewer #1: All comments have been addressed

Reviewer #3: All comments have been addressed

2. Is the manuscript technically sound, and do the data support the conclusions?

The manuscript must describe a technically sound piece of scientific research with data that supports the conclusions. Experiments must have been conducted rigorously, with appropriate controls, replication, and sample sizes. The conclusions must be drawn appropriately based on the data presented.

Reviewer #1: Yes

Reviewer #3: Yes

3. Has the statistical analysis been performed appropriately and rigorously?

Reviewer #1: Yes

Reviewer #3: (No Response)

4. Have the authors made all data underlying the findings in their manuscript fully available?

The [PLOS Data policy](http://www.plosone.org/static/policies.action#sharing) requires authors to make all data underlying the findings described in their manuscript fully available without restriction, with rare exception (please refer to the Data Availability Statement in the manuscript PDF file). The data should be provided as part of the manuscript or its supporting information, or deposited to a public repository. For example, in addition to summary statistics, the data points behind means, medians and variance measures should be available. If there are restrictions on publicly sharing data—e.g. participant privacy or use of data from a third party—those must be specified.

Reviewer #1: Yes

Reviewer #3: No

5. Is the manuscript presented in an intelligible fashion and written in standard English?

PLOS ONE does not copyedit accepted manuscripts, so the language in submitted articles must be clear, correct, and unambiguous. Any typographical or grammatical errors should be corrected at revision, so please note any specific errors here.

Reviewer #1: Yes

Reviewer #3: Yes

6. Review Comments to the Author

Please use the space provided to explain your answers to the questions above. You may also include additional comments for the author, including concerns about dual publication, research ethics, or publication ethics. (Please upload your review as an attachment if it exceeds 20,000 characters)

Reviewer #1: Manuscript is written is of good relevant information. The importance of understanding the relation between sarcopenia, TUG, pneumonia, balance test is expalined.

Reviewer #3: Dear authors,
I appreciate your efforts in taking into account the feedback provided by the reviewers, and for diligently addressing the suggestions and comments. This commitment enhanced the quality of the manuscript.

But one final comment remains, is to provide a clear justification or reference for choosing the cutoff point for the TUG test.

**Response)** Thank you for your valuable comments. Our study used data from a special health-screening program called the National Screening Program for Transitional Ages (NSPTA) provided by the National Health Insurance Service-Senior Cohort (NHIS-Senior) database of the Korean National Health Insurance (KNHI) service. The NSPTA includes a questionnaire regarding mental/cognitive function and depression screening, as well as physical function tests (TUG test, unipedal balance test) that cover common problems of the elderly, such as frailty.

In the Korean NSPTA, the TUG test cutoff is defined as normal at 10 seconds or less and abnormal at 10 seconds or more based on expert opinion. In addition, the cutoff of 9-10 seconds is used as a cutoff in various populations, and the cutoff of 10 seconds is commonly used in ethnically similar Asian countries such as Japan and Singapore *[Reference- J Am Med Dir Assoc. 2021 Aug;22(8):1640-1645., Phys Ther 2017;97(4):417-24.]* In fact, most Korean studies that have analyzed the association between TUG test and clinical outcomes using data from the National Screening Program for Transitional Ages in Korea have also used a cutoff value of 10 seconds for the TUG *test [Reference- Bone. 2019 Oct;127:474-481., BMC Geriatr . 2020 Mar 19;20(1):111., J Korean Med Sci. 2020 Jan 20; 35(3): e25., J Gerontol A Biol Sci Med Sci. 2018;73(9):1238-43.].*

Therefore, as you commented, we have further described and written the rationale for setting the cutoff in the Independent variable section as follows. (Line 157-164)

We would also like to express our sincere thanks to the editor and reviewers for their helpful comments to improve the paper.

**Materials and methods**

**…**

**Independent variable**

…

*The cut off value of the TUG test varies depending on the study, but the cut off value of 9-10 seconds is used in various population groups (20, 21) , and the cut off value of 10 seconds is also used in Asian populations such as Japan and Singapore (22, 23). In fact, most studies investigating the association of the TUG test with clinical outcomes such as dementia, fractures, and functional disability in Korean populations have used a 10-second cut off value (24-27). Based on these existing studies and expert opinions, the Korean NSPTA defines a TUG test of less than 10 seconds as normal, so in our study, participants were divided into two groups based on their TUG test (<10 seconds as normal and ≥10 seconds as slow gait).*

7. PLOS authors have the option to publish the peer review history of their article ([what does this mean?](https://journals.plos.org/plosone/s/editorial-and-peer-review-process#loc-peer-review-history)). If published, this will include your full peer review and any attached files.


If you choose “no”, your identity will remain anonymous but your review may still be made public.


**Do you want your identity to be public for this peer review?** For information about this choice, including consent withdrawal, please see our [Privacy Policy](https://www.plos.org/privacy-policy).

Reviewer #1: **Yes:** PREM KUMAR BHOJARA

Reviewer #3: **Yes:** Sumayeh Abujaber
